# Tackling noise pollution in climate adaptation and mitigation: planetary health benefits towards a net-zero future

**DOI:** 10.1093/oxfclm/kgaf021

**Published:** 2025-08-20

**Authors:** Yutong Samuel Cai, Jing Huang, Enock Havyarimana, Anna L Hansell

**Affiliations:** Centre for Environmental Health and Sustainability, University of Leicester, Leicester, LE1 7RH, United Kingdom; NIHR Leicester Biomedical Research Centre, Leicester General Hospital, Leicester, LE5 4PW, United Kingdom; Institute for Environmental Futures, University of Leicester, Leicester, LE1 7RH, United Kingdom; NIHR Health Protection Research Unit in Chemical Threats and Hazards at the University of Leicester, Leicester, LE1 7RH, United Kingdom; Department of Occupational and Environmental Health Sciences, School of Public Health, Peking University, Beijing, 100191, China; Centre for Environmental Health and Sustainability, University of Leicester, Leicester, LE1 7RH, United Kingdom; NIHR Leicester Biomedical Research Centre, Leicester General Hospital, Leicester, LE5 4PW, United Kingdom; Centre for Environmental Health and Sustainability, University of Leicester, Leicester, LE1 7RH, United Kingdom; NIHR Leicester Biomedical Research Centre, Leicester General Hospital, Leicester, LE5 4PW, United Kingdom; Institute for Environmental Futures, University of Leicester, Leicester, LE1 7RH, United Kingdom; NIHR Health Protection Research Unit in Chemical Threats and Hazards at the University of Leicester, Leicester, LE1 7RH, United Kingdom

**Keywords:** noise pollution, climate change, net-zero policy, planetary health, one health

## Abstract

Noise pollution is a planetary health problem. This perspective article sets out to provide a high-level summary of recent scientific evidence on the impacts of noise pollution from transport on human and the natural environment. Beyond annoyance and sleep disturbance, evidence has indicated that traffic noise is associated with cardiovascular diseases, metabolic outcomes, mental health and neurological health. Current estimates of the burden of ill health due to noise pollution are likely to underpredict the true impact as newer evidence emerges. Furthermore, current net-zero policy discussions tend to be dominated by strategic priorities such as population mobility, economic growth, and air pollution. Noise is often overlooked or only considered after problems arise. We explore the intersections between noise pollution and climate strategies relating to transport, natural environment, housing and building, and offer insights into some of the potential benefits and caveat to human health, in a planetary health perspective.

## Introduction

Over the last 15 years, health impacts and disease burden relating to environmental noise pollution, particularly that from the transport sector, have been increasingly researched. In 2011, a World Health Organization (WHO) report suggested that at least one million healthy life years are lost every year from traffic noise in west Europe [1]. Since then, many studies, the majority of which were conducted in Europe, have indicated that traffic noise pollution is a risk factor for a wide-range of health impacts, from cardiovascular and metabolic disease, to dementia and mortality. Some recent published burden-of-disease (BoD) calculations have clearly signalled that traffic noise pollution is of growing concern to public health. In England, 40% of all adults are chronically exposed to road traffic noise level exceeding 50 decibels(dB) of L_den_ (A-weighted average sound pressure level over all days, evenings and nights in a year with an evening weighting of 5 dB and a night weighting of 10 dB), a level above which adverse health effects are likely to occur [2]. In 2018 alone, noise pollution from road, rail and air traffic respectively led to an estimated loss of 97 000, 13 000, and 17 000 healthy life years across England. Among the four Nordic capital cities (Oslo, Helsinki, Copenhagen, and Stockholm), loss of healthy life years ranged from 329 to 485 per 100 000 population for road traffic noise, and from 44 to 146 per 100 000 for railway traffic noise in 2017 [3]. Across the European region, 48 000 incident heart diseases and 12 000 premature deaths are estimated to occur annually due to traffic noise pollution [4]. Calculations based on strategic noise mapping of the harmonised European Environmental Noise Directive framework are almost certainly underestimated, as they consider major sources of noise only [2]. In low-and-middle-income countries (LMICs), evidence is scarce on BoD from traffic noise pollution. However, given the large number of populations exposed to high-to-very high traffic noise levels in LMICs and the underlying health conditions of some of the world’s most vulnerable populations [5], the BoD estimates are likely much larger than what was seen in Europe. For example, in Tehran, Iran, about 697 healthy life years per 100 000 population was lost in 2017 due to exposure to significantly high levels of road traffic noise [6], which is over 40% higher than that reported in Nordic countries [3].

While it is true that these BoD estimates are subject to further updates with emerging evidence, they have collectively suggested that the adverse public health impacts of noise pollution from transport deserve more recognition. In addition, potential health impacts of other forms of environmental noise (e.g. wind turbine, construction) are also reported [7, 8]. Focused attention is urgently needed, not only to advance the scientific research agenda, but also to advocate noise pollution as an important planetary and public health issue in the current debates on policymaking, especially in the net-zero contexts as we move towards a more sustainable, and healthier 2050. In July 2023, a report published by the House of Lords Science and Technology Committee has urged the UK Government to plan early on about the possible implications on noise pollution as the country implements net-zero strategy and climate adaptation programmes up to 2050 [9]. Several targets of the net-zero strategies, as reported in the UK and many other countries, including transport decarbonization, clean energy, greener building, better natural environment, are closely linked to noise pollution. However, as we combat climate change and transition to a net-zero future, there is little attention across the policy landscapes about the delicate health benefits and risks that are associated with noise pollution.

This perspective article presents a reflection on this topic by exploring the health impacts of noise pollution, with a main focus on traffic noise, and the implications from current and future climate adaptation and mitigation on noise pollution, bearing in mind a planetary health perspective considering interconnectedness and thinking holistically in terms of challenges and solutions [10]. We need to re-emphasize the significance of noise pollution on the health of human and the environment, and consider that tackling noise pollution should be an integral component in climate actions to protect planetary health.

## Impacts of transport noise pollution on human health

**Figure 1.**
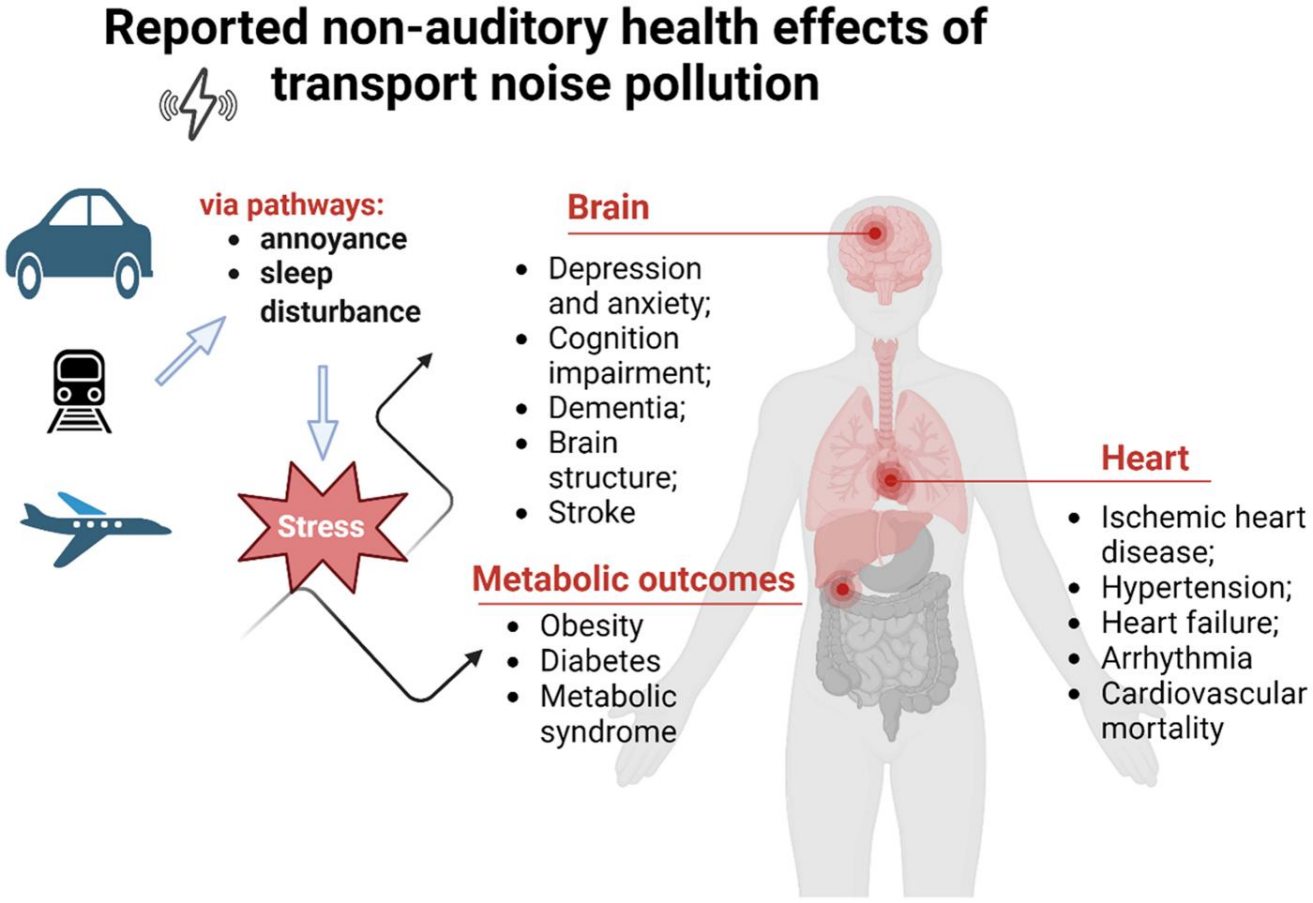
Non-auditory health effects of transport noise pollution.

The health impacts of both chronic and acute exposure to transport noise pollution have been increasingly reported in different study populations over the last decade. While noise-induced annoyance and sleep disturbance contribute to a significant part of current BoD calculations, evidence base for other important health outcomes in adults is strengthening (Fig. 1). We herein provide an up-to-date brief summary of latest scientific evidence since the published WHO reviews in 2018 [11].

### Cardiovascular and metabolic health

Transport noise pollution has been linked to prevalence, incidence and mortality of a range of cardiovascular diseases (CVD) and their intermediate risk factors, with most studies available for road traffic noise. In a recent umbrella review, road traffic noise pollution was identified as a risk factor for ischemic heart diseases (IHD), stroke and heart failure, with the relative risk (RR) increased by 4.1% (95% CI: 2.3–59), 4.6% (95% CI: 1.3–81) and 4.4% (95% CI: 1.7–71) per 10 dB(A) higher of annual average road noise respectively [12]. The risk of all incident CVD events increased by 3.2% (95% CI: 1.1–52) per 10 dB(A) higher of annual average road noise. Road traffic noise was also found to be a risk factor for CVD mortality (RR: 1.05, 95% CI: 1.02–107). There are a smaller number of studies investigating CVD impacts of rail and air traffic noise so more uncertainty around effect estimates as published by WHO in 2018 [11], but more recent cohort studies support similar impacts on CVD risk (own works, https://www.isesisee2025.org/program/ Abstract number #2575).

There are very few studies on short-term effects of noise [13]. In Madrid, each decibel higher same day exposure to diurnal road noise was significantly associated with 0.3% (95% CI: 0.2–0.5%) increase in risk for CVD emergency hospital admissions [14]. The first two reports relating to aircraft noise, both of case-crossover design, found that acute exposure during night-time was associated with cardiovascular mortality and morbidity. The Swiss National Cohort study found that in the 2 h preceding cardiovascular death, those exposed to the highest night-time aircraft noise (>50 dB) had a 44% (odds ratio (OR): 1.44, 95% CI: 1.03–204) increased risk, as compared to those exposed to the reference level (<20 dB) [15]. A study of aircraft noise from London Heathrow found associations with CVD hospital admissions for previous night exposure, but did not find associations with CVD mortality possibly due to much smaller numbers of cases [16]. For both studies, it was suggested that these observed associations were partly related to sleep disturbance during night, and that high night-time noise variability was associated with higher risks.

High blood pressure is one of the most important risk factors for CVD. Many previous cross-sectional studies have found an association between road traffic noise exposure and high blood pressure [17]. While these studies are useful for hypothesis generation, by design they cannot explore whether road traffic noise exposure leads to development of high blood pressure over time. A recent study from UK Biobank has provided important insights into this [18]. In a study sample of over 240 000 individuals who were free of hypertension at baseline, it was found that over a 8-year of follow-up, every 10 dB higher of 24-h road traffic noise exposure (L_den_) was significantly associated with a 7% (95% CI: 2–13%) increased risk of incident hypertension. Again, there are very few studies investigating the associations with either rail or aircraft noise exposure for incident hypertension, therefore further investigations are urgently warranted. A Swedish study of 4854 men and women reported an HR of 1.16 (95% CI: 1.08–1.24) per 10 dB L_den_ between aircraft noise and hypertension incidence [19]. Recently, two women-only cohort studies in the United States found no [20] or weak [21] associations.

Obesity and diabetes are the two most common metabolic outcomes. Associations have been seen with transport noise exposures, supported by plausible mechanistic pathways related to stress response and sleep disturbance [22]. These studies provide further evidence that transport noise exposures can lead to development of CVD partly via adverse changes in metabolic risk factors, such as obesity and diabetes. In general, current evidence seems to support a positive association between road traffic noise and several adiposity markers, with the association with central obesity (measured by waist circumference) being the most consistent across the studies [23]. A 2022 systematic review pooled data from 10 cohort studies of 4 994 171 participants and 417 332 Type 2 diabetes cases in the meta-analysis [24]. The review found that risk of incident diabetes increased by 6% (RR: 1.06, 95% CI: 1.03–1.09) per 10 dB higher of 24-h road traffic noise. A recent study and updated meta-analysis from the Swiss National Cohort reported similar findings when combining incidence and mortality data [25]. However, the majority of included studies were rated as likely having high risk of bias, while results across studies presented high heterogeneity.

### Brain health

Some recent evidence has suggested that traffic noise pollution is a potential risk factor for incident dementia and cognitive impairment among older adults. In terms of incident dementia, the largest study to date is the national cohort study in Denmark [26], of nearly two million participants aged 60 years and older, which included 103 500 incident dementia cases during a 14-year of follow-up. Both road and rail traffic noise exposures were significantly associated with incidence of all-cause dementia and subtypes particularly Alzheimer’s disease. Findings from our own investigation in the UK Biobank cohort echoed those reported in the Danish study. We observed a 15% (95% CI: 2.2%- 29.4%) increase in incident Alzheimer’s disease for every 10 dB higher exposure of road traffic noise (L_den_) [27]. Other studies of smaller sample sizes have reported no associations [28, 29]. A registry-based cohort study in London reported that the weak association between night-time road noise and incident dementia was likely confounded by air pollution [30]. More recently, the Danish Nurse Cohort study reported that a statistically significant association between road traffic noise and incident dementia appeared to be confounded by PM_2.5_. Study of noise pollution and adverse changes in brain morphology is still in its infancy. Further studies with a much larger sample size are required to confirm if there is an association with transport noise and dementia and if so, which brain structural and/or functional markers are more predictive of noise pollution exposure [31, 32].

In terms of cognitive decline, a Swedish cohort study of 2594 participants aged 60 years and above who had been followed for up to 16 years, it was found that exposure to aircraft noise was significantly associated with a faster annual rate of decline in global cognitive function score [33]. In addition, both aircraft noise and railway noise contributed significantly to the development of CIND (cognitive impairment, no dementia). There was no association of road traffic noise with cognitive decline. Another study of 1612 Mexican-American participants based in the US found that road noise was weakly associated with CIND, however, this association was lost after adjusting for local air pollution [28]. The small number of other studies available from Europe, of similar sample sizes, have also consistently observed that road traffic noise was not associated with cognitive function or impairment [34], or that the association was likely confounded by air pollution [35] or a chance finding [36] due to multiple testing.

### Mental health

Traffic noise has long been recognised as a risk factor for annoyance [37, 38]. Across the European Union, in 2017, at least 18 million people were highly annoyed by exposure to unhealthy traffic (road, rail and aircraft) noise level above 55 dB(A) L_den_ [39]. A health impact assessment study across 724 cities in Europe estimated that at city level the median value of the proportion of the populations being highly annoyed by road traffic noise was 7.6% (Interquartile Range (IQR): 5.6–118) [37]. Different traffic noise appeared to have differential impacts on annoyance. Several studies have indicated that aircraft noise seems to be more annoying [40–42], followed by railway and road noise. The degree of intermittency (i.e. contribution of individual noise events above background levels) of noise can have a marked difference in annoyance reaction that is different for different types of noise. For example, a low 24-h intermittency ratio (IR) of road traffic noise, related to longer intervals between noise events, seems to trigger stronger annoyance reactions, as compared to high 24-h IR of road noise [42]. The opposite is seen for aircraft and rail noise where higher intermittency is associated with higher annoyance.

A 2020 systematic review of studies published up to December 2019 found that long-term aircraft noise exposure was significantly associated with increased depression risk by 12% (95% CI: 2–23) per 10 dB higher of noise [38]. In contrast, the effect sizes for railway and road noise were between 2 and 3%. In addition, the review also reported that road noise exposure was associated with anxiety at a magnitude similar to depression risk. Studies of anxiety related to other sources of noise pollution are generally lacking. Since 2020, there have been a handful of traffic noise studies published with mixed results [43–46]. Among these, a study in Switzerland reported that both road and aircraft noise may increase the risk of incident depression via noise annoyance [43]. Recently, a novel study in the Swiss National Cohort reported that road and railway noise was associated with a higher risk of suicide, with hazard ratio (HR) (95% CI) per 10 dB higher of L_den_ being 1.040 (95% CI: 1.015–1065) and 1.022 (95% CI: 1.004–1041) [47]. Very few studies investigated the short-term or acute impacts from traffic noise on mental health. A case-time series study in Switzerland found that over the lag period of 3 h after exposure to military aircraft noise, the odds of medication administration for psychiatric patients were significantly increased [48]. In Madrid, each decible higher same day noise exposure (largely road noise) was associated with 0.8% (95% CI: 0.3–1.3%) increase in total admissions due to mental disorders [49].

Both noise annoyance and noise sensitivity (an independent predictor of the annoyance response to environmental noise) may play a key role in the relationship between noise pollution and mental health outcomes [50]. In a study of 2398 men in England, noise sensitivity was linked to a higher risk of psychological ill-health when exposed to road traffic noise [51]. High noise annoyance has been linked to several mental health outcomes including depression, anxiety and poor general mental health [52]. There are also studies reporting that noise annoyance and noise sensitivity, particularly that from aircraft traffic, may act as mediators, and/or modifiers, on the associations between noise exposures and many physical health outcomes such as CVD [53, 54]. For example, in a pooled analysis from seven European cohorts, aircraft noise annoyance and aircraft noise sensitivity each significantly associated with increased risks of antihypertensive medication use [53].

### Other health outcomes

In the past few years, a few studies have reported the epidemiological associations between traffic noise pollution and other less-studied health outcomes. In the Danish Nurse Cohort Study, long-term exposure to road traffic noise was associated with respiratory disease mortality [55], and development of chronic obstructive pulmonary disease (COPD) [56]; both associations appeared to be independent of air pollution. These findings however will need to be validated in other study populations and that underlying biological mechanisms should be elucidated. There is some suggestive evidence that road or railway traffic noise exposure may link to development of certain cancer types, including colon cancer [57], breast cancer [58] and Hodgkin lymphoma [59]. Again, studies remain very few and more studies are warranted to draw a conclusion. Several possible biological mechanisms are underlying the link between long-term traffic noise exposure and cancer development. Noise-related sleep disturbance may cause disruptions to circadian rhythms, which subsequently suppress melatonin, an anti-carcinogenic agent [60]. In addition, stress responses following noise exposures cause oxidative stress and inflammation, which are key pathways in carcinogenesis [61].

## Impacts of noise pollution on the nature

Noise pollution is not only a threat to human health but also a threat to natural resources, for example, marine and terrestrial wildlife [62]. It is well known that excessive, high-level anthropogenic noise can disturb animals to various extents, affecting their distributions, behaviour, reproductive process, emigration and even mortality, which all will ultimately affect ecological communities [63]. In a planetary health perspective, such changes to natural systems will have consequences on human health [10]. For example, human multi-sensory experience with nature may be compromised, as people see and hear less wildlife in a dominated anthropogenic soundscape (defined as all audible sounds in a specific area). This will negatively impact psychological ecosystem services and mental health [64, 65]. Biodiversity loss has been linked to noise pollution [66], which will negatively affect human health partly via reduced restoring capacities [67]. Both positive experience with nature and increased biodiversity are important health-promoting aspects for the general public, in particular those living in urban areas where noise pollution is a main environmental stressor.

## Tackling noise pollution and climate change: a planetary health thinking

Climate change is one of the greatest health challenges in the 21st century. As current evidence suggests, in the short to medium term, its direct and indirect impacts on human health mainly operate via increasing vulnerability of environmental factors (e.g. ambient temperature, air quality, vector distributions, drinking water, food, biodiversity), social factors (e.g. livelihoods, access to healthcare) and health system capacity and resilience. In the long term, such health impacts would largely depend on whether, and the extent to which, bold climate action is taken. The recent *Lancet Countdown 2023 report* has called for a people-centred transformation on climate policy agenda [68].

Given that the world is urbanizing at an unprecedented rate, more populations will be exposed to unhealthy noise levels if policy progress on noise pollution was inadequate. Currently, countries across the world are taking steps to combat climate change through various ambitious initiatives. Some of these initiatives are focusing on co-benefits on human health by tackling air pollution and climate change together [69]. Air pollution is the single biggest environmental risk factor globally [70], and is closely interconnected with climate change. In contrast, despite commonly co-existing with air pollution due to similar sources, noise pollution has far less been mentioned in those initiatives. It is necessary to raise the profile of noise pollution in the current and future climate adaptation and mitigation plans. Addressing climate change will unlock many health benefits for both human and the environment in part through better management of noise at a population level.

### Decarbonization of transport

Transport, particularly that from road vehicles, remains the largest emitting sector of greenhouse gas (GHG) emissions in the UK and across Europe [71]. In addition to emissions of GHG and other air pollutants (e.g. particulate matter, nitrogen oxides, etc.), transport also causes noise pollution. One of the key objectives of many governmental net-zero strategies on transport is reducing the use of motorised transport by promoting public transport and active travel (i.e. a modal shift from driving to cycling and walking). This decarbonization pathway has a wide range of benefits to the health of both human and the environment. A 2018 review from then Public Health England (now UK Health Security Agency) has reported that the benefits of cycling and walking on both physical and mental health in the populations could potentially save the healthcare system billions of pounds [72]. It is anticipated that an ambitious investment in public transport facilities and active travel infrastructure will result in improved human health partly via reductions in both air and noise pollution [73]. For example, a study into low-traffic neighbourhood (LTN) in London, UK, found that the scheme can potentially lead to 6% to 9% reductions of local air pollution and traffic volumes [74]. In Temple Cowley, a residential area of Oxford, UK, a novel study involving eight low-cost audio sensors investigated the impacts of LTN implementation on urban noise [75]. Urban noise was measured before and after the LTN implementation in terms of acoustic energy and source apportionment (anthropogenic or biotic), with the latter characterised based on noise frequency using Normalised Difference Soundscape Index (NDSI). Markedly, after the introduction of the LTN, the study found that among the eight locations, almost all locations experienced a reduction in acoustic energy, except one which was likely a displacement location. Meanwhile, all locations except for the control location experienced more biotic sounds, showing that the LTN can reduce the amount of human-generated noise. This study clearly demonstrated the positive impacts of LTN on urban noise, although moving forward it will be beneficial to measure noise levels in absolute terms in residential areas and assess the resulting health impacts. Whilst more traffic intervention studies are on the horizon to assess their impacts on noise levels, in the meantime, modelling studies of different scenarios could provide some important initial insights to guide decision-making.

Another key objective in decarbonizing road transport lies in the electrification of road vehicles fleets. In the UK and European Union (EU), the sale of new petrol and diesel cars is currently set to be phased out from 2030 and 2035 onwards respectively, hence a substantial increase in electric vehicles (EVs) on the roads is expected. In fact, the global market share of EVs has risen rapidly in recent years and will continue to rise in the foreseeable future [76]. EVs are generally regarded as a desirable approach to cut combustion-related air pollutants. They are in general also quieter, producing noise at almost half the level of those from petrol and diesel cars. Given this, for pedestrian safety, the European Union have implemented a new law requiring all EVs to install a sound system to generate sound at around 56 dB when travelling at 20 km/h [77], which is above the current health-based level (53 dB) set by the WHO guidelines [11]. A simulation study indicated that whilst a mass adoption of EVs in the future will improve environmental acoustic conditions, it seems the benefits are marginal if travel speed is above 50 km/h in urban settings, and that a 1-2dB reduction may be seen at a speed of 30 km/h if there was no presence of heavy vehicles [78]. This is in line with another modelling study which concluded that noise from light EVs at certain frequencies are sensitive to travel speed and acceleration [79]. A recent modelling study in Hong Kong found that whilst a full adoption of electric cars in the city only resulted in a 1–2 dB reduction of traffic noise overall, a full adoption of electric bus fleets would reduce traffic noise levels up to 4 dB [80]. This finding highlights a greater potential of traffic noise reduction through electrifying buses and/or other heavy-duty vehicles in a compact city, that may potentially bring health benefits. These studies highlighted the importance of considering trade-offs between cutting GHG emissions and noise pollution when devising and prioritizing net-zero policy on different types of EVs to maximise population health benefits [81]. A recent review has summarised all the studies investigating the impacts of mass transition to EVs on urban noise [82]. In brief, across the studies, it was shown that introducing EVs can cut environmental noise by 1–5 dB(A) at low speeds and frequencies. Such reductions can still have potential to lower health and economic costs due to noise-related illness on population level. Overall, from a policy perspective, electrifying the current vehicle fleets may not necessarily achieve optimal traffic noise reduction on the roads. It has been suggested that other interventional measures such as low-noise tyres and road surface, as well as noise barriers for compact residential areas in close proximity to traffic hotspots should also be implemented and legally regulated alongside the mass transition to EVs [80, 83].

Another important GHG emitter in the transport sector, but which is more challenging to decarbonise is the aviation industry. Currently, policy debates focus on reducing carbon emissions by improving fuel efficiency via air traffic management etc., as well as supporting the development of sustainable, low-carbon fuels and aircrafts [84]. Whilst the investment in developing technologically advanced aircrafts (e.g. full electric, hybrid-electric, turbo-electric aircrafts) sounds desirable in tackling GHG emissions, how efficient this will be in leading to reductions in noise pollution remains to be further investigated [85, 86]. For example, some preliminary modelling studies found that electric aircraft are likely to reduce noise pollution levels near airports during take-off but increase noise levels towards landing [85]. More research and investment are needed in the near future to ensure that technological advancements for aircraft noise reduction progresses in parallel with GHG reduction in the sector.

### Preserving natural environment

Natural environments such as green and blue spaces provide multiple benefits to human and the environment. Preserving and restoring the natural green world is an integrated part of net-zero strategy in order to massively sequester carbon emission. This also has implications in reducing exposure to harmful levels of noise pollution, particularly in the cities. Green space is regarded to act as a buffer against noise pollution and also a source of natural sound [87]. A recent systematic review of studies investigating the impacts of green space composition (e.g. area) and configuration (e.g. shape, distribution, connectivity) on urban noise has provided some insightful policy-relevant evidence [88]. Among the 23 reviewed studies, consistent associations were found between green space composition and configuration and decreased environmental noise levels to various extents. Specifically, greater total area, larger or clustered sets of patches, taller trees, and deeper vertical vegetation were all associated with greater noise reductions. Research suggested that people living closer to green space infrastructure or in a greener environment, were less sensitive to, and annoyed, by noise, although this could be a masking effect of rustling leaves for example [89]. The pathways through which green infrastructure (open grass, parks, woodlands, forests, etc.) mitigates noise pollution remain to be further explored. Mass planting of trees has been highlighted as a mean to combat GHG emissions in many net-zero policies. It should be noted that different tree characteristics (species, bark, crown, leaf, trunks, height) have varying degrees of noise absorption, also considering proximity to noise sources and the size of green infrastructure [87]. Soundscape has rarely been discussed in current net-zero policy on green environment. Of note, the Welsh Government recently published a pioneering piece of soundscape legislation to protect nature soundscape while also considering wider environmental factors such as air quality [90].

Compared to green spaces, blue spaces, or aquatic environments, have been less researched in terms of their health impacts and therefore less mentioned in policy-making. Apart from studies of underwater noise and marine wildlife, the interaction between noise and blue spaces on human health requires further investigations before any intervention is implemented. Nevertheless, experimental studies have generally shown stress-reducing impacts of water-based sounds [91]. However, its long-term impact, in particular when in combination with those from traffic noise, remains unknown in terms of human health.

### Greener energy, building and housing

Residential building and housing sector are key GHG emitters. This is mainly due to the use of natural gas for heating and cooking. The use of solid wood for residential heating is also becoming a concern in recent years in high-income countries [92]. Enhancing the building and housing design to improve household energy performance has been frequently mentioned in net-zero strategies. Currently, policies focus on reducing energy waste in existing house stocks (insulation, installation of double-glazed windows, etc.) as well as investment on new, greener buildings which are built to be fit-for-purpose for the changing climate. Apart from better energy performance, better-sealed homes also offer benefits such as indoor thermal comfort, as well as lower exposure to harmful noise pollution from roads outside. There is, however, a trade-off with indoor air quality, therefore ventilation is still key to mitigate poor indoor air quality.

It is still poorly understood regarding the fine balance between household energy performance, noise pollution from home indoor or outdoor and individuals’ behaviours such as window-opening, use of mechanical ventilation or Air Source Heat Pumps (ASPHs). For example, mechanical ventilation seems to be effective in reducing indoor air pollution and mould, however, during night-time ventilation noise could significantly impact sleep quality [93] or cause annoyance [94]. In the UK, noise from mechanical ventilation systems is not legally regulated, although the regulation rules have suggested that for bedroom and living room, noise from mechanical ventilation systems should not exceed 30 dB [95]. This limit, however, does not account for individual’s noise sensitivity [94]. ASPHs, as a low-carbon technology, is a key player of the UK Government’s Warm Homes Plan to cut GHG emissions [96]. However, if poorly installed or designed, ASPHs could be a residential noise source [97]. To date, impacts of noise from ASPHs on both physical and mental health among the residents, both short-to-medium term and long-term, remain unknown. Research is ongoing regarding how to reduce noise from heat pumps [98]. In addition, both mechanical ventilation systems and ASPHs tend to be noisier when they age. Robust evidence is warranted to inform future guidelines and practices on the design, maintenance, and use of mechanical ventilations and ASHPs, and to understand their associated health impacts.

## Addressing noise pollution through environmental and climate policy making

Across the world, including the United Kingdom, policy progress on noise mitigation and strategies is gathering its pace in recent years. We refer readers to the literature on these policies, best practices and guidelines that are produced and regulated by international organizations and national governments [99, 100]. We advocate that policy that aims to mitigate noise pollution should consider planetary health as a key cornerstone, which will ultimately improve quality of life and support sustainable development. From a UK perspective, we crafted a few thoughts that may be helpful to advance the policy agenda in this area. First, noise or soundscape, should be part of the broader environmental legislation to protect the environment and promote healthy living. A good example is the soundscape legislation brought in by the Welsh Government [90]. Such environmental regulations and legislations should adopt a one health and lifecycle view of our living environment, considering multiple environmental stressors including air pollution and GHG, noise, green infrastructure and biodiversity, chemical pollution and any emerging hazards [101]. In addition, these regulations and legislations should also be enforceable at both national and local government policy levels. Second, we need a holistic view of current and future net zero policies to avoid unintended consequences. For example, noise generated by air-sourced heat pump in dense urban residential areas may cause concerns among residents and therefore may negatively impact on the mass roll-out of this low-carbon technology.

## Conclusion

Noise pollution is a planetary health problem and its impacts on human disease burden should not be overlooked. In fact, noise pollution has been identified as an issue that links up all the United Nations (UN) Sustainable Development Goals (SDGs) [102]. Given that the world is urbanizing rapidly, failure to mitigate noise pollution may potentially jeopardise the achievement of SDGs, which ultimately will impact on planetary health and the wider society as a whole. As we move towards a net-zero future across the world, this provides an opportunity to address noise pollution in parallel with carbon emissions and air pollution in current policy discussion and decision-making. Climate adaptations and mitigations could offer a range of benefits to both planetary and public health, in part via substantial reductions of exposure to environmental stressors including noise pollution.

## Data Availability

No new data were generated or analysed in support of this research.
